# P-453. Invasive Infections by Streptococcus pyogenes in the Pediatric Population of Chile: A Multicenter Study

**DOI:** 10.1093/ofid/ofaf695.668

**Published:** 2026-01-11

**Authors:** Mirta Acuña, Marcela Zúñiga, Yennybeth Leiva, María C Rivacoba, Stephania Passalaqua, Armando Lavayen, Lily Contreras, Ana M Espinoza, Antonella Capdeville

**Affiliations:** Roberto del rio children hospital, Santiago, Region Metropolitana, Chile; Roberto del Río Hospital and University of Chile, Santiago, Region Metropolitana, Chile; University of Chile, Santiago, Region Metropolitana, Chile; Exequiel Ganzález Cortés Hospital, Santiago, Region Metropolitana, Chile; Osorno Hospital, Osorno, Los Lagos, Chile; Sotero del Río Hospital, Santiago, Region Metropolitana, Chile; Carabineros's Hospital, Santiago, Region Metropolitana, Chile; University of Chile, Santiago, Region Metropolitana, Chile; University of Chile, Santiago, Region Metropolitana, Chile

## Abstract

**Background:**

*Streptococcus pyogenes* is a common cause of pediatric infections, ranging from mild to life-threatening. In December 2022, teh UK reported an unusual rise in *S. pyogenes* infections, including invasive forms. The phenomenon spread globally, prompting a WHO alert due to increased cases and severity. In Chile,a similar trend began in 2024. This study aimed to describe the epidemiological and clinical characteristics of invasive *S. pyogenes* infections in Chilean children.

**Methods:**

A retrospective, multicenter study was conducted across five hospitals in Chile in pediatric population between january 2024 and march 2025. Medical records of patients with invasive S. pyogenes infections were reviewed, collecting demographic, clinical and microbiological data. Descriptive statistics were used for data analysis.

**Results:**

82 cases were included in 5 hospitals: Roberto del Río (51.2%), Exequiel González Cortés (22.0%), Sótero del Río (14.6%), Osorno (7.3%) an Carabineros (4.9%). Ages ranged from 6 months to 14 years (mean 7 years and 9 month); 57.3% were male and 61% had no underlying conditions. Of those with comorbilities, 34.4% had chronic respiratory disease. Viral coinfections were identified in 28% (e.g., rhinovirus, influenza A/B, RSV, metapneumovirus).

Clinically, 40.2% had scarlatiniform rash; 53.7% presented with toxic/septic shock. Most frecuent foci were scarlet fever (24.4%), pulmonary anosteoarticular (20.7% ech). Mean hospital stay was 13.4 days; 68.3% requiered ICU (mean 8 days). Case fatality was 3.7% (3 patients).

Etiology was confirmed mainly by cuture (89%), folowed by rapid testing (26.8%) and molecular methods (2.4%). Isolation sites included pharynx, blood, jooints, pleura and CSF.

Surgical management was performed in 54.9%. All patient recived antibiotics, mostly penicillin or third-generation cephalosporins associated with clindamycin (79.3%). IVIG was used in 36.6%, corticosteroids in 17.1%.

**Conclusion:**

This is the first collaborative survillance of invasive S.pyogenes in Chilean pediatric patients. Most were previously healthy males. One-third had viral coinfections. Severe presentations were common, with high ICU use. Fatality rate reached 3.7%

**Disclosures:**

All Authors: No reported disclosures

